# Laser Needle Knife's Effects on Rabbits Cervical Spondylopathy of Vertebral Artery, Fibrinogen, and Blood Viscosity

**DOI:** 10.3389/fsurg.2022.778608

**Published:** 2022-04-11

**Authors:** Zhenyu Huang, Siwei Xie, Fang Liu, Ting Zhang, Yiwen Gu

**Affiliations:** ^1^Department of Acupuncture, Hangzhou Red Cross Hospital, Hangzhou, China; ^2^The Second Clinical Medical College, Zhejiang Chinese Medical University, Hangzhou, China; ^3^College of Medical Technology, Zhejiang Chinese Medical University, Hangzhou, China

**Keywords:** laser needle-knife, cervical vertebral artery, acupuncture, fibrinogen, blood viscosity

## Abstract

**Objective:**

To determine the effect of laser needle-knife on vertebroarterial morphology, fibrinogen and blood viscosity in a rabbit model of cervical spondylotic arteriopathy (CSA) and the mechanism of action involved.

**Methods:**

A number of 40 healthy common grade rabbits were divided into four groups: normal control, model, acupuncture, and laser needle knife group. The normal control group does not establish a CSA rabbit model, and the other groups all establish a CSA rabbit model, but they are treated in different ways. CSA model rabbits were treated with acupuncture and moxibustion at “fengchi” and “cervical Jiaji” points, rabbits in the laser needle knife group were treated with “Jiaji” points, and the acupuncture points were punctured with the laser needle knife. The location of the acupuncture points is determined according to the acupoint map of the experimental map. The right vertebroarterial morphology before and after the treatment was analyzed by scanning electron microscope, and FIB concentration and blood viscosity were determined using the coagulation method.

**Results:**

After the treatment, the capillary and micropore hyperplasia in the laser needle knife group were more evident than that in the model group. Acupuncture and laser needle knife therapy can reduce whole blood viscosity (1/s, 5/s), and that the distinction between the two treatments is not statistically evident.

**Conclusion:**

Acupuncture and laser needle knife can regulate the coagulation and fibrinolysis system in CSA, stimulate capillary and micropore hyperplasia, reduce blood viscosity, and improve blood circulation, which may be one of the therapeutic mechanisms behind the laser needle knife treatment of CSA.

## Introduction

Cervical spondylosis vertebral arteriopathy (CSA) is a familiar and frequently occurring disorder in middle-aged and elderly people, which accounted for 20–30% of cervical spondylosis cases. The incidence rate of CSA is the second only to cervical radiculopathy and mainly manifests as dizziness. The cause is due to insufficient blood supply to the basilar artery. CSA can also cause changes in hematological properties, which leads to microcirculatory disorders, increased blood viscosity, decreased fibrinolytic activity, and platelet accumulation. Traditional Chinese Medicine (TCM) classifies CSA under the category of “dizziness” and “acute necrosis of bone” and is believed to be caused by the insufficient blood and chi, with the weakness in both the liver and kidney, which results in insufficient blood supply to the vertebral artery (1–3). The onset of cervical spondylosis is not only related to age, sex, occupation, but also related to work and lifestyle factors, such as high pillow sleeping, long-term use of computer and mobile phones, working at the desk, the lack of physical exercise, and neck fatigue history (4, 5). The patients with cervical spondylosis tends to be recurrent (6, 7). In addition, the patients with cervical spondylosis show mental tension, anxiety, and insomnia (8, 9). At the same time, the cervical spondylosis may have some interactions with spinal disease (10). CSA can also lead to the presence of memory dysfunction in model rats, which may be due to vertebrobasilar insufficiency resulting in damage to the hippocampus (11). CSA patients generally have the problem of cervical instability and displacement. Attention should be paid to maintaining the stability of cervical spine during treatment. In addition, from the data analysis, the degree of displacement of the cervical spine and vertebral artery type of cervical spondylosis of vertebral basilar artery blood flow velocity between the most relevant (12). In a recent study, the researchers found that the pathogenesis is closely related to the serum thromboxane (TXA_2_) and endothelin (ET), and the mechanism of action of TXA_2_ and ET is involved in the vertebral artery vasoconstriction and spasm (13).

Starting from the theory of liver-based therapy on CSA, each syndrome type was treated with TCM prescription accordingly, which can make that most of the clinical symptoms were remarkably reduced, and the clinical effect is better than western medicine. It can be said that the treatment of CSA is reasonable and effective. Compared to those who were taking flunarizine hydrochloride capsules, the treatment through the TCM has advantages of the lower rate of adverse reaction and the lower rate of recurrence (14). Modified Banxia Baizhu Tianma decoction can improve the vertebral artery elasticity, make the elasticity of blood vessels increase, can improve red cell activity, inhibit erythrocyte aggregation, and improve blood flow of vertebral artery through the unit of time; the effect of this aspect is basically equivalent to that of Jinfukang granule (15). Tongduhuoxue decoction can accelerate the rabbit with CSA of shenduxuyu syndrome artery and basilar artery blood flow velocity, can reduce whole blood and plasma viscosity, improve blood supply of vertebral artery, improve concentration and blood sticky situation, and elevate cyclic adenosine monophosphate (cAMP)/cyclic guanosine monophosphate (cGMP) ratio and plasma adrenocorticotropic hormone (ACTH) content, so as to improve deficiency pathological role. Additionally, Tongduhuoxue decoction can elevate the rabbit with CSA of shenduxuyu syndrome serum NO, ET level reduced by adjusting the vasoactive factors, improve the state of systolic and diastolic blood vessels, reduce the rabbit with CSA of shenduxuyu syndrome disc nucleus of prostaglandin E2 (PGE2) content, reduce the disc inflammatory mediators, and delay the disc degeneration (16).

The facts have proved that acupuncture therapy of “nape seven needle” can remarkably improve the symptoms and function, and the clinical efficacy of patients, and can improve the patient's basilar artery, left vertebral artery, and right vertebral artery mean blood flow velocity, can make culpability index, reduce resistant index, and improve cerebral blood supply. The treatment of CSA by the acupuncture therapy of “nape seven needle” is accurate, economical, and convenient, safe, and reliable (17). Small needle knife through suboccipital muscles loses solution treatment suboccipital muscles myofascial pain syndrome (MPS) has remarkable curative effect. At the same time, it can effectively treat vertebral artery atlanto-axial period of soft tissue factors of CSA, which obviously improve the symptoms such as dizziness and headache (18). It was found that digital-pressing manipulation at Dazhui with spoon needle could reduce the expression of the tumor necrosis factor-alpha (TNF-α) and interleukin 1 beta (IL-1β) in chondrocytes possibly through the regulation of MKK3/6-p38MAPK signaling pathway, so as to reduce the inflammatory reaction in chondrocytes to delay the degeneration of cervical intervertebral disc (19).

The He-Ne laser needle knife provides a combination of the surgical effect of a small needle knife and the biological effects of a He-Ne laser to loosen tissue adhesion. He-Ne lasers can increase the permeability of cells and blood vessel walls, which leads to the improved cellular metabolism and enhanced vitality, which are conducive to the dissipation of tissue edema and the absorption of hematomas, thereby relieving symptoms and restoring the function of the body (20–24).

The above studies provide the important parameters for the He-Ne laser needle knife treatment of CSA and are of great significance for the development of the laser needle knife treatment.

## Methodology

### Experimental Animals and Model Construction

In the experiment, 40 healthy adult rabbits (male and female in half) were used. The average weight of each rabbit is 2.5 ± 0.2 kg. The rabbits were housed in the Experimental Animal Center of Zhejiang University of Chinese Medicine. The rabbits were live at the right temperature and moderation and were provided the right amount of food and water. The model of Hu et al. provides a reference for the establishment of the model (25).

### Grouping and Treatment

In the experiment, there are 40 rabbits randomly divided into 4 groups of 10 each. The four groups are the normal control group (N group), model group (M group), acupuncture group (A group), and laser needle knife group (L group). N group: The rabbits were immobilized during treatment and did not induce CSA. M group: The rabbits were immobilized during treatment and induce CSA. A group: at 2 weeks post-CSA induction, the rabbit's treatment was started. Fengchi point and cervical Jiaji points (C3–C7) are chosen to be the acupuncture points. In the process of acupuncture, experimenters insert filiform needles (1.5 inches) into the above-mentioned six points. When the needles were inserted, the experimenter made sure that all the needles remained on the body for 30 min, thus making appropriate adjustments every 10 min. The rabbits were treated for every 2 days. The number of treatments was 10. After 20 days of treatment, the rabbits were painted with color Doppler ultrasound. After all the hormones, the rabbit is euthanized. L group: at 2 weeks post-CSA induction, the rabbit's treatment was started. During the treatment, the rabbit is attached to the surgical container and its cervical vertebrae are bent. The treatment target of this experiment comes from the acupuncture point map of the experimental rabbit. The selected treatment target is Jiaji points on both sides of C5 rotation. It should be noted that before the treatment, disinfection is necessary. In this experiment, iodine and phosphorus disinfectants should be used to disinfect the skin surface. The 1% lidocaine solution is a good anesthetic, and the researchers used it to anesthetize the rabbit. The researchers used a laser needle to cut, peel, and loosen the skin until it was free of nodules and blockages. The power radiated by He-NE laser is 200 mW. Each stimulation was maintained for 30 min and was administered once for every 10 days for a total of three times.

### SEM Images of Different Samples

The samples of a gold coating under vacuum conditions were installed on the copper column with conductive double-sided tape and evaluated using a scanning electron microscope (HITACHI SU1510).

### Analytical Methods

The weight of rabbits from each group was taken 1 day before the model establishment, 14 days after the model establishment (prior to treatment), 24 days after the model establishment (after 10 days treated), and 34 days after the model establishment (after 20 days of treatment). Blood was taken from the rabbit's ear vein and collected in a tube containing sodium citrate as the anticoagulant. The volume collected was about 5 ml. FIB concentration and blood viscosity were determined using the coagulation method. The spirit and manner (eyelid and physical movements), fur luster, nose and tail color, feces, hunching, and feeding behavior of all rabbits were observed daily and recorded once for every 3 days starting from the day of CSA induction. The enzyme-linked immunosorbent assay (ELISA) was performed according to the manufacturer's instructions [PI3K (CK-E72396), p-AKt (CK-E72395), VEGF (CK-E72257), HIF1α (CK-E72097), and mTOR (E72180, MLBio)], and the results were analyzed using a SpectraMax Plus 383 long-wavelength microplate reader (MD) and SoftMax Pro 5.4.1. The PI3K, Akt, mTOR, HIF-1α, and VEGF levels in rabbit's vertebral arteries were determined according to the manufacturer's instructions. The data were organized into an experimental result database and statistically analyzed using SPSS version 17.0 software (SPSS, Chicago, IL, USA). The data were expressed as the mean ± standard deviation ($\bar{x}\pm$ s). *p* < 0.01 or *p* < 0.05 was considered as statistically significant.

## Result Analysis

### Effect of Laser Needle Knife Treatment on the Weight of CSA Rabbits

**Figure 1:**
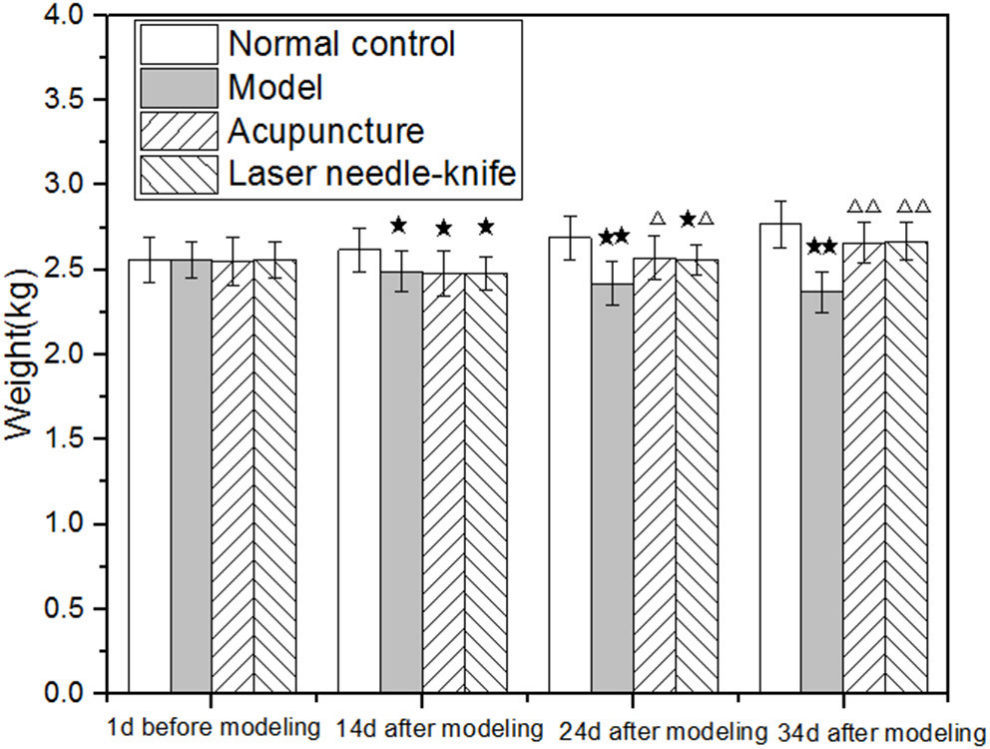
Weight of CSA rabbits. Compared with normal control group, *P < 0.05, **P < 0.01; Compared with model group, ^Δ^*P* < 0.05, ^ΔΔ^*P* < 0.01.

As the experiment progressed, the weight of rabbits in the N group showed a gradually increasing trend. After 14 days of the model establishment (before treatment), the weight of the rabbits in N group, that is, the weight of the non-onset rabbit group is significantly higher than the group of three diseased rabbits (*p* < 0.05). After 24 days of the modeling (after 10 days of treatment), the weight of rabbits in the *N* age group was remarkably greater than that in the M (*p* < 0.01) and L groups (*p* < 0.05). It is hard to find some differences between the weight of the A and the N groups (*p* > 0.05). After 34 days of the model establishment (after 20 days of treatment), the body weight of rabbits in the M group was substantially lower than that of those in the N group (*p* < 0.01). It is hard to find some distinct differences between the A and the L groups (*p* > 0.05). Compared with M group, groups A and L are healthier. This judgment comes from the greater weight gain of these two groups of rabbits (both *p* < 0.01) and is listed in Figure 1.

### Effects of Laser Needle Knife Treatment on Morphology of the Vertebral Artery of CSA Rabbits

**Figure 2:**
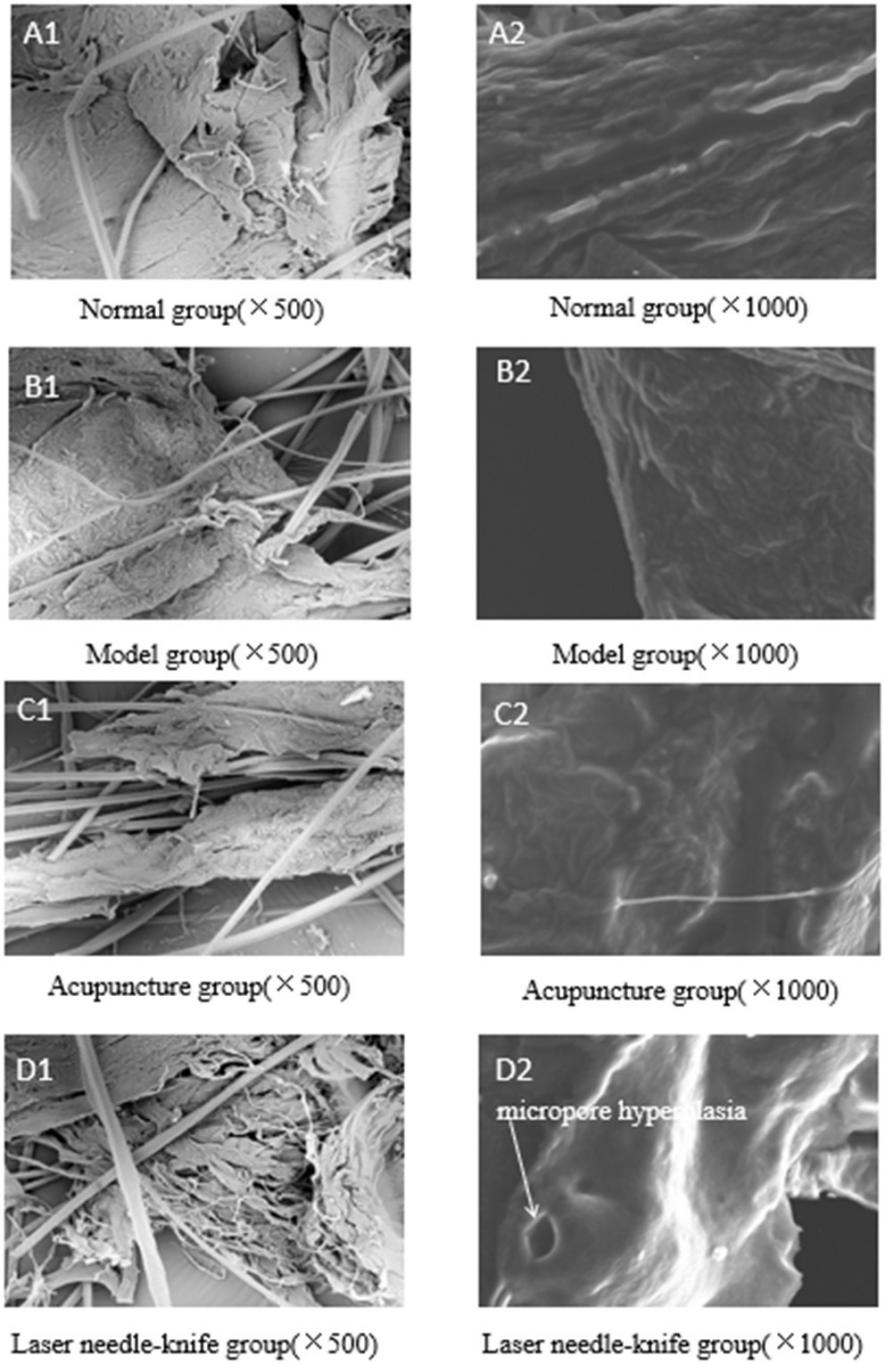
**(A–D)** SEM images of different samples.

After treatment, the surface morphology of vertebral arteries of rabbits in each group is shown in Figure 2. As shown in Figure 2A, there are many folds and folds on the surface of the acellular matrix in the rabbit's vertebral artery. Additionally, these cases are more common in *N* group. Folds are beneficial to the health of mice, which often indicates that the vertebral artery is good without CSA and can better complete the task of nutritional transport. In addition, compared with N group, rabbits in M group had poorer vascular conditions, with more arterial occlusions and fewer folds on their surface, which was detrimental to the health of mice because it showed that CSA caused a decrease in blood flow and insufficient nutrition for transportation (Figure 2B). After treatment in the A group, the level of capillary proliferation on the vertebral artery surface was low (Figure 2C). This suggests that the treatment improves microcirculation and blood flow and alleviates CSA symptoms. The rabbits in the L group had more capillary proliferation on the vertebral artery surface than the rabbits in the A group, and rabbits in L group can be observed by the observation that the number of micropores on the surface and cross-section of the vertebral artery is more than that in M group (Figure 2D). The above evidence indicates that laser needle knife is treated by increasing the number of micropores in the treatment of CSA. Considering that the micropores are conducive to the delivery of nutrients.

### Effect of Laser Needle Knife Treatment on FIB Concentrations in CSA Rabbits

Compared with N group, from the observed situation, the plasma FIB of M group rabbits is larger, as shown in Table 1 (*p* < 0.01), while the plasma FIB from the rabbits in the A and L groups showed no statistically distinct difference (*p* > 0.05). The plasma FIB of rabbits in M group is reasonably high, which is significantly higher than that in A and L groups (*p* < 0.01), which suggests that two treatments can reduce plasma FIB concentration, and the difference between the two treatments is not evident.

**Table 1:** Fib concentrations in CSA rabbits ($\bar{x}\pm$ S) (Mg /Dl).

**Group**	**Number of cases**	**Fib concentration**
Normal control group	10	234 ± 71
Model group	10	295 ± 46^**^
acupuncture group	10	229 ± 52^ΔΔ^
Laser needle-knife group	10	222 ± 61^ΔΔ^

*Compared with normal control group*.

** *P < 0.01*;

*Compared with model group*,

ΔΔ *P < 0.01*.

### Effect of Laser Needle Knife Therapy on Hemorheological Indexes of CSA Rabbits

The hemorheology indicators from each group after treatment are shown in Table 2. Whole blood viscosity (1/s): Compared with the N group, the M group is better during the observation period in the test item of whole blood viscosity only (*p* < 0.05), and under the predictable conditions, thew indicators of whole blood viscosity in groups A and L have been repeatedly tested and found that the indicator of whole blood viscosity is generally lower than that in M group, probably due to the therapeutic effect (*p* < 0.05). The A, L, and M groups may have a condition with total blood viscosity distinctly lower than the N group under the effect of treatment (*p* < 0.05), while the acupuncture and the L group showed substantially lower whole blood viscosity than the M group (*p* < 0.05). Whole blood viscosity (30/s and 200/s) did not show statistical differences (*p* > 0.05). These data suggest that acupuncture and laser needle knife therapy can reduce whole blood viscosity (1/s, 5/s), and that the difference between the two treatments is not statistically distinct. The plasma concentration was tested between the two groups. Under the results of multiple tests, no significant difference was found between the two groups (*p* > 0.05). The reduced viscosity of whole blood (low cut): the overall blood viscosity of the L group was higher than that of the N group (*p* < 0.05), and after multiple tests of the whole blood viscosity of L group, it can be found that the whole blood viscosity index is generally lower than that of M group (*p* < 0.05). Reduced viscosity of whole blood (high cut): The M and A groups had substantially higher whole blood viscosity than the N group (*p* < 0.05), while the acupuncture and the whole blood viscosity of L group are lower than the M group (*p* < 0.05). In the experiment, Carson viscosity in the M group was higher than that in the N group (*p* = 0.05). However, it cannot be found an obvious difference in Carson viscosity between the three groups except the N group (*p* = 0.05).

**Table 2:** Hemorheology indicators($\bar{x}\pm$s) (m·Pa/s).

**Indicators**	**Normal control group**	**Model group**	**Acupuncture group**	**Laser needle-knife group**
**Whole blood viscosity**
1/s	11.42 ± 1.60	14.71 ± 1.30^*^	12.36 ± 1.28^Δ^	12.44 ± 1.82^Δ^
5/s	4.97 ± 0.52	6.26 ± 0.46^*^	5.50 ± 0.38^*^^Δ^	5.28 ± 0.50^*^^Δ^
30/s	3.02 ± 0.27	4.04 ± 0.38	3.60 ± 0.39	3.51 ± 0.53
200/s	2.67 ± 0.47	3.25 ± 0.53	2.80 ± 0.36	2.78 ± 0.40
Plasma viscosity	1.26 ± 0.08	1.38 ± 0.60	1.35 ± 0.08	1.28 ± 0.23
Whole blood viscosity, low-shear	21.29 ± 1.88	25.29 ± 2.45^*^	22.37 ± 2.86	23.64 ± 2.19^*^
Whole blood viscosity, high-shear	3.38 ± 0.71	4.65 ± 1.42^*^	3.63 ± 1.06^*^^Δ^	3.48 ± 0.65^Δ^
Caron viscosity	2.30 ± 0.32	2.80 ± 0.65^*^	2.39 ± 0.27	2.57 ± 0.41

*Compared with normal control group*.

* *P < 0.05*;

*Compared with model group*,

Δ *P < 0.05*.

### Effect of Laser Needle Knife on Levels of PI-3K, AKt, mTOR, HIF-1α, and VEGF in CSA Rabbits

Post-treatment PI-3K levels in the right cervical artery of rabbits per group are shown in Table 3. PI-3K levels were significantly lower in the model group compared to the normal control group (*p* < 0.05), but similar between acupuncture, laser needle knife, and normal control groups (*p* > 0.05). In addition, PI-3K levels were significantly higher in the acupuncture and laser needle knife groups compared to the model group (both *p* < 0.05), but was similar between the two treatment groups (*p* > 0.05). Post-treatment p-AKt levels in the right cervical artery of rabbits per group are shown in Table 4. p-AKt concentration was significantly lower in the model group than in the normal control group (*p* < 0.05), but was similar between the acupuncture, laser needle knife, and normal control groups (*p* > 0.05). p-AKT concentration was significantly higher in the acupuncture and laser needle knife groups than in the model group (both *p* < 0.05), but was similar between the two treatment groups (*p* > 0.05). Post-treatment mTOR levels in the right cervical artery of rabbits per group are shown in Table 5. The mTOR concentration was significantly lower in the model group compared to the normal control group (*p* < 0.05), but was similar between acupuncture, laser needle knife, and normal control groups (*p* > 0.05). Moreover, the mTOR concentration was significantly higher in the acupuncture and laser needle knife groups compared to the model group (both *p* < 0.05), but was similar between the two treatment groups (*p* > 0.05). Post-treatment HIF-1α levels in the right cervical artery of rabbits per group are shown in Table 6. HIF-1α levels were significantly lower in the model group compared to the normal control group (*p* <0.05), but was similar between acupuncture, laser needle knife, and normal control groups (*p* > 0.05). In addition, HIF-1α levels were significantly higher in the acupuncture and laser needle knife groups when compared to the model group (*p* < 0.01 and *p* < 0.05, respectively), but were similar between the two treatment groups (*p* > 0.05).Post-treatment VEGF levels in the right cervical artery of rabbits per group are shown in Table 7. The VEGF levels were significantly lower in the model group compared to the normal control group (*p* < 0.05), but were similar between acupuncture, laser needle knife, and normal control groups (*p* > 0.05). Moreover, VEGF levels were significantly higher in the acupuncture and laser needle knife groups compared to the model group (both *p* < 0.05), but were similar between the two treatment groups (*p* > 0.05).

**Table 3:** Comparison of PI-3K content in CSA Rabbits ($\bar{x}\pm$ S) (pg/mg).

**Group**	**Number of cases**	**PI-3K content**
Normal control group	10	249.97 ± 47.26
Model group	10	211.54 ± 31.37^*^
Acupuncture group	10	254.38 ± 50.29^Δ^
Laser needle-knife group	10	263.18 ± 46.99^Δ^

*Compared with normal control group*.

* *P < 0.05*;

*compared with model group*,

Δ *P < 0.05*.

**Table 4:** Comparison of p-AKt content in CSA Rabbits ($\bar{x}\pm$ S) (ng/mg).

**Group**	**Number of cases**	**p-AKt content**
Normal Control Group	10	1.84 ± 0.36
Model Group	10	1.52 ± 0.30^*^
Acupuncture Group	10	2.15 ± 0.60^Δ^
Laser Needle-Knife Group	10	2.17 ± 0.62^Δ^

*Compared with normal control group*.

* *P < 0.05*;

*Compared with model group*,

Δ *P < 0.05*.

**Table 5:** Comparison of mTOR content in CSA Rabbits ($\bar{x}\pm$ S) (ng/mg).

**Group**	**Number of cases**	**mTOR content**
Normal control group	10	22.46 ± 4.42
Model group	10	18.06 ± 4.64^*^
Acupuncture group	10	25.52 ± 6.29^ΔΔ^
Laser needle-knife group	10	26.69 ± 5.63^ΔΔ^

*Compared with normal control group*,

* *P < 0.05*;

*Compared with model group*,

ΔΔ *P < 0.01*.

**Table 6:** Comparison of HIF-1α content in CSA rabbits ($\bar{x}\pm$ S) (pg/mg).

**Group**	**Number of cases**	**HIF-1α content**
Normal control group	10	9.11 ± 2.04
Model group	10	7.42 ± 1.38^*^
Acupuncture group	10	9.80 ± 2.01^ΔΔ^
Laser needle-knife group	10	9.58 ± 2.31^Δ^

*Compared with normal control group*,

* *P < 0.05*;

*Compared with model group*,

Δ *P < 0.05*,

ΔΔ *P < 0.01*.

**Table 7:** Comparison of VEGF content in CSA Rabbits ($\bar{x}\pm$ S) (pg/mg).

**Group**	**Number of cases**	**VEGF content**
Normal control group	10	79.40 ± 13.49
Model group	10	65.14 ± 13.15^*^
Acupuncture group	10	85.13 ± 20.20^Δ^
Laser needle-knife group	10	89.59 ± 25.62^Δ^

*Compared with normal control group*.

* *P < 0.05*;

*Compared with model group*,

Δ *P < 0.05*.

## Conclusions

As an important coagulation factor in the body, increase in FIB concentration increases blood viscosity, stimulates platelet aggregation, and leads to the formation of blood clots that can block arterial blood flow, which is a factor that needs special consideration in CSA. Acupuncture and laser needle knife can regulate the coagulation and fibrinolysis system in CSA, reduce blood viscosity, and improve blood circulation, which may be one of the therapeutic mechanisms behind laser needle knife treatment of CSA. This combination therapy has many advantages, such as long effect, easy to operate, save time and strength, safe, and clean.

## Data Availability Statement

The raw data supporting the conclusions of this article will be made available by the authors, without undue reservation.

## Ethics Statement

The animal study was reviewed and approved by Zhejiang Chinese Medical University, 58 Binwen Road, Hangzhou 3100053, China.

## Impact Statement

In the rabbit model of CSA, the therapeutic mechanism of laser needle knife has been discussed by the perspective of vertebral body morphology, FIB, and blood viscosity. The results show that acupuncture and laser needle knife can regulate the coagulation and fibrinolysis system in CSA, stimulate capillary and micropore hyperplasia, reduce blood viscosity, and improve blood circulation, which may be one of the therapeutic mechanisms behind the laser needle knife treatment of CSA. This combination therapy has many advantages, such as long effect, easy to operate, save time and strength, safe, and clean. In this work, we found that angiogenesis is the part worth continuing to study in CSA treatment by providing abundant oxygen and nutrients to vertebral artery which eventually influence the flow velocity of blood.

## Author Contributions

ZH and SX conducted the experiments. FL and TZ supplied critical animals. ZH, SX, and YG wrote the manuscript. All the authors participated in the design, interpretation of the studies, analysis of the data, and review of the manuscript.

## Funding

This work was supported by from National Natural Science Foundation of China (No. 81704144), Chinese Medicine Research Program of Zhejiang Province (No. 2020ZB190), the Fifth National Research Program of TCM Clinical Talents in State Administration of Traditional Chinese Medicine (Chinese Medicine Teaching Leter [2022] No. 1), and Zhejiang Province Health High-level Personal Training Project (Zhejiang Health Office [2020] No. 18).

## Conflict of Interest

The authors declare that the research was conducted in the absence of any commercial or financial relationships that could be construed as a potential conflict of interest.

## Publisher's Note

All claims expressed in this article are solely those of the authors and do not necessarily represent those of their affiliated organizations, or those of the publisher, the editors and the reviewers. Any product that may be evaluated in this article, or claim that may be made by its manufacturer, is not guaranteed or endorsed by the publisher.
